# Reproducibility, reliability and validity of population-based administrative health data for the assessment of cancer non-related comorbidities

**DOI:** 10.1371/journal.pone.0172814

**Published:** 2017-03-06

**Authors:** Camille Maringe, Helen Fowler, Bernard Rachet, Miguel Angel Luque-Fernandez

**Affiliations:** Cancer survival group, Department of Epidemiology and Population Health, London School of Hygiene and Tropical Medicine, London, United Kingdom; Universiti Sains Malaysia, MALAYSIA

## Abstract

**Background:**

Patients with comorbidities do not receive optimal treatment for their cancer, leading to lower cancer survival. Information on individual comorbidities is not straightforward to derive from population-based administrative health datasets. We described the development of a reproducible algorithm to extract the individual Charlson index comorbidities from such data. We illustrated the algorithm with 1,789 laryngeal cancer patients diagnosed in England in 2013. We aimed to clearly set out and advocate the time-related assumptions specified in the algorithm by providing empirical evidence for them.

**Methods:**

Comorbidities were assessed from hospital records in the ten years preceding cancer diagnosis and internal reliability of the hospital records was checked. Data were right-truncated 6 or 12 months prior to cancer diagnosis to avoid inclusion of potentially cancer-related comorbidities. We tested for collider bias using Cox regression.

**Results:**

Our administrative data showed weak to moderate internal reliability to identify comorbidities (ICC ranging between 0.1 and 0.6) but a notably high external validity (86.3%). We showed a reverse protective effect of non-cancer related Chronic Obstructive Pulmonary Disease (COPD) when the effect is split into cancer and non-cancer related COPD (Age-adjusted HR: 0.95, 95% CI:0.7–1.28 for non-cancer related comorbidities). Furthermore, we showed that a window of 6 years before diagnosis is an optimal period for the assessment of comorbidities.

**Conclusion:**

To formulate a robust approach for assessing common comorbidities, it is important that assumptions made are explicitly stated and empirically proven. We provide a transparent and consistent approach useful to researchers looking to assess comorbidities for cancer patients using administrative health data.

## Background

When modelling cancer survival in population-based research, it is relevant to account for potential confounders and effect modifiers, such as comorbid conditions, frequently linked to clinically relevant outcomes.[1, 2] Most studies found that cancer patients with comorbidity had poorer survival than those without comorbidity.[1] The presence of comorbidities may delay or favour a timely cancer diagnosis.[3–5] In addition, it has been hypothesised that patients with comorbidities do not receive standard cancer treatments such as surgery, chemotherapy, and radiation therapy as often as patients without comorbidities.[1] Thus, the use of individual comorbidities or comorbidity scores such as the Charlson index[6] will enrich our understanding of differences in cancer survival outcomes in observational population-based studies.

Comorbidities are defined as the coexistence of disorders, in addition to a primary disease of interest, which are causally unrelated to the primary disease (e.g. cancer).[7, 8] A myriad of comorbidity indices have been developed, some more specifically for cancer patients (simple condition, counts of simple conditions, weighted indices and organ-based system).[9]

The Charlson comorbidity index (CCI) is the most extensively studied and most widely used comorbidity index in the medical literature.[10] The widespread use of this index could be explained by the fact that it is not designed for patients with a particular disease and is recommended when overall mortality is the outcome of interest.[10] It does not require extensive information, which makes it appealing to researchers who access administrative data rather than individual clinical notes.[9] However, no gold standard approach to measure comorbidity in the context of cancer exists, and the source of data to ascertain comorbidities varies. [9] Two main sources of data are commonly used to ascertain comorbidities: clinical records and administrative data. In population-based research, administrative data has been suggested as the best available option to ascertain comorbidities and predict in-hospital mortality or 6-month mortality for the CCI.[9, 10]

A report regarding the administrative sources of data used for deriving comorbidities showed a lack of consistency, validity, and replicability of a broad majority of the studies deriving comorbidities.[9] Furthermore, the studies describing the comorbidity index did not offer a clear description of the underlying assumptions made to obtain the algorithm nor provide the code used, thereby limiting the opportunity to assess and replicate the work. Consequently, researchers can make differing assumptions in their evaluation of comorbidities, leading to conflicting findings.

We aimed to construct a robust algorithm that is both transparent and replicable to assess comorbidities using population-based hospital administrative data. First, we described and evaluated the assumptions underlying the development of an algorithm, using the hospital episode statistics (HES) in England for the period 2003–2013. We then evaluated the internal and external validity and quality of these data to extract and use comorbidity information.

## Materials and methods

### Study design, data and linkage strategy

We developed a retrospective longitudinal assessment of comorbidities for cancer patients diagnosed in England during 2013. Information on cancer patients with a malignant invasive primary tumour was obtained from cancer registrations in England. This contains patient and tumour variables including relevant dates (birth, diagnosis, last vital status), sex, age at diagnosis, deprivation, cancer site and morphology. We used population-based administrative hospital discharge data for the assessment of comorbid conditions. Namely, we analysed Hospital Episode Statistics (HES) data,[11] including accident and emergency (A&E), inpatient and outpatient data streams in England for the period 2003 to 2013. HES contains clinical, administrative, and demographic information about individual patients. The diagnostic information uses the International Classification of Diseases (10th revision) (ICD-10) [12] and operations are coded using the Office of Population Censuses and Surveys Surgical Operations and Procedures (4th edition) (OPCS-4).[13]

HES data had been linked to the cancer registrations from Public Health England using a deterministic linkage strategy based on an individual ID (NHS number), date of birth, sex and postcode.

### Data management

#### Overall assumptions

Overall, we assumed that HES is a valid source of data for the assessment of comorbidities at a population-based level. However, the evaluation of comorbidities depends heavily on both age and probability of attending the hospital (outpatient/inpatient) in the years preceding the cancer diagnosis. Given the chronic aspect of comorbidities, we also assumed that once a comorbidity is recorded in HES, the patient suffers from that comorbidity up until the time of cancer diagnosis. To explain and evaluate our algorithm, we focussed on patients diagnosed with laryngeal cancer.

#### Algorithm

**Fig 1:**
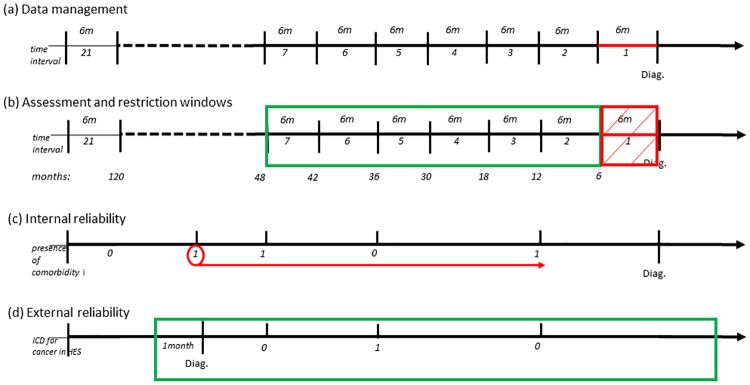
Graphical representation of data management and time-related assumptions.

From HES, we selected all 14 diagnostic variables containing ICD-10 diagnosis codes[12] (version 4) for each episode registered. The time scale refers to time pre-cancer diagnosis, which we split into six-monthly intervals (Fig 1A). We compared the hospital episode start date to the cancer diagnosis date to confirm its inclusion in the different time intervals. Each interval was examined independently. Each diagnosis field was scanned for the 17 co-morbid conditions that compose the CCI (listed in S1 Table) and morbid obesity.[14] If a comorbidity (*i*) was recorded in a given six-month interval (*j*), we updated the corresponding binary indicator variable (x_*ij*_ = 1). The assessment of comorbidities for periods longer than six months were simply the aggregation of the information contained in all binary variables derived for each six-monthly interval, assuming that once the comorbidity was identified it was just counted once. We also retained the episode date at which a comorbidity was first recorded. We consider the patient as the unit of analysis.

#### Time-related assumptions

Minimising the potential for selection bias when assessing comorbidities requires the development of an algorithm that will evaluate the same amount of person-time at risk for any given patient included in the assessment. It allows each patient to have the same probability of being diagnosed with comorbidities in relation to the time under assessment. Table 1 shows the minimum number of years, for each cohort of patients, for which we can assess comorbidities. We used the 2013 cohort as a reference to which we compared the comorbidity information derived from shorter time windows. Given this data constraint, we had to consider carefully the optimal time window for the assessment of comorbidities based on the trade-off between long HES history and the number of cancer patient cohorts to evaluate.

**Table 1:** Data structure of the cancer registrations linked to HES records for the evaluation of comorbidities.

	Cancer registrations																															
	1971…2003	2004			2005			2006			2007			2008			2009			2010			2011			2012			2013			
		*potential follow-up (years)*			*potential follow-up (years)*			*potential follow-up (years)*			*potential follow-up (years)*			*potential follow-up (years)*			*potential follow-up (years)*			*potential follow-up (years)*			*potential follow-up (years)*			*potential follow-up (years)*			*potential follow-up (years*			
Restriction window:		-	*6m*	*12m*	-	*6m*	*12m*	-	*6m*	*12m*	-	*6m*	*12m*	-	*6m*	*12m*	-	*6m*	*12m*	-	*6m*	*12m*	-	*6m*	*12m*	-	*6m*	*12m*	-	*6m*	*12m*	
*Hospital Episodes Statistics*																																
**2003**		**1**	*0*.*5*	0	**2**	*1*.*5*	*1*	**3**	*2*.*5*	*2*	**4**	*3*.*5*	*3*	**5**	*4*.*5*	*4*	**6**	*5*.*5*	*5*	**7**	*6*.*5*	*6*	**8**	*7*.*5*	*7*	**9**	*8*.*5*	*8*	**10**	*7*.*5*	*9*	**2003**
**2004**		**0**			**1**	*0*.*5*	*0*	**2**	*2*.*5*	*1*	**3**	*2*.*5*	*2*	**4**	*3*.*5*	*3*	**5**	*4*.*5*	*4*	**6**	*5*.*5*	*5*	**7**	*6*.*5*	*6*	**8**	*7*.*5*	*7*	**9**	*8*.*5*	*8*	**2004**
**2005**					**0**			**1**	*0*.*5*	*0*	**2**	*1*.*5*	*1*	**3**	*2*.*5*	*2*	**4**	*3*.*5*	*3*	**5**	*4*.*5*	*4*	**6**	*5*.*5*	*5*	**7**	*6*.*5*	*6*	**8**	*7*.*5*	*7*	**2005**
**2006**								**0**			**1**	*0*.*5*	*0*	**2**	*1*.*5*	*1*	**3**	*2*.*5*	*2*	**4**	*3*.*5*	*3*	**5**	*4*.*5*	*4*	**6**	*5*.*5*	*5*	**7**	*6*.*5*	*6*	**2006**
**2007**														**1**		*0*	**2**	*1*.*5*	*1*	**3**	*2*.*5*	*2*	**4**	*3*.*5*	*3*	**5**	*4*.*5*	*4*	**6**	*5*.*5*	*5*	**2007**
**2008**																	**1**	*0*.*5*	*0*	**2**	*1*.*5*	*1*	**3**	*2*.*5*	*2*	**4**	*3*.*5*	*3*	**5**	*4*.*5*	*4*	**2008**
**2009**																				**1**	*0*.*5*	*0*	**2**	*1*.*5*	*1*	**3**	*2*.*5*	*2*	**4**	*3*.*5*	*3*	**2009**
**2010**																				**0**			**1**	*0*.*5*	*0*	**2**	*1*.*5*	*1*	**3**	*2*.*5*	*2*	**2010**
**2011**																							**0**			**1**	*0*.*5*	*0*	**2**	*1*.*5*	*1*	**2011**
**2012**																										**0**			**1**	*0*.*5*	*0*	**2012**
**2013**																													**0**			**2013**
**2014**																																**2014**

Number in cell: minimum number of years available for assessment of comorbidities for patients diagnosed in the index year. Three scenarios are considered: no restriction window (no), 6 (6m) or 12 (12m) months restriction windows, during which comorbidities are not assessed.

Furthermore, considering the definition of comorbidity as the occurrence of disorders which are causally unrelated to cancer, we defined comorbidities identified shortly before diagnosis as cancer-related (Fig 1B). Thus there is a risk of a collider bias given that cancer-related comorbidities may be a common effect of exposure and outcome, and contradictory associations may arise between non-cancer related comorbidities and cancer survival. [15] To mitigate the possibility of selection bias we created restriction windows of 6, 12 or 24 months before the cancer diagnosis, during which comorbidities first registered were excluded. However, cancer-related comorbidities may be of interest in studies aiming to evaluate factors associated with the cancer treatment decision. In this particular case, the restriction mentioned above will not apply.

### Validation and statistical analyses

First, to evaluate the optimal time window for the retrospective assessment of comorbidities, we compared the cumulative incidences of comorbidities for the 2013 cohort of laryngeal cancer patients using consecutive time restrictions, and showed the corresponding percentages of comorbidities lost.

Second, we used two semi-parametric Cox proportional hazard models to estimate the age-adjusted effect of non-cancer related comorbidities on cancer survival. The first model did not differentiate cancer- and non-cancer-related comorbidities. Then, the effect of comorbidities on cancer survival was compared with a second model where cancer and non-cancer related comorbidities were modelled as independent variables. For both models, we fitted three different versions relating to various lengths of the restriction window (6, 12 and 24 months).

Finally, to measure the reliability and consistency of HES to assess comorbidities we computed the intraclass correlation coefficient (ICC) [16] and calculated the percentage of agreement between two independent sources for cancer diagnosis information, namely HES and the cancer registrations.[17] We defined internal reliability as the extent to which two or more successive HES episodes for any given patient report identical or additional comorbidities (Fig 1C).[18, 19] We used non-linear generalised random effects models to derive the ICC for each of the 17 CCI conditions and their respective 95% CI. The external validity of HES was defined following the Centre for Disease Control (CDC) surveillance strategy for the assessment of reliability between two different sources of data.[20, 21] Cancer registrations were considered as the gold standard for cancer diagnosis. The HES diagnostic fields were screened for a laryngeal cancer diagnostic code from 1 month before the cancer registry diagnosis date (Fig 1D). Then we estimated the percentage of agreement for cancer diagnosis between the two sources and derived 95%CI based on the exact test.[17]

All data management and statistical analyses were performed using STATA version 14.

## Results

**Fig 2:**
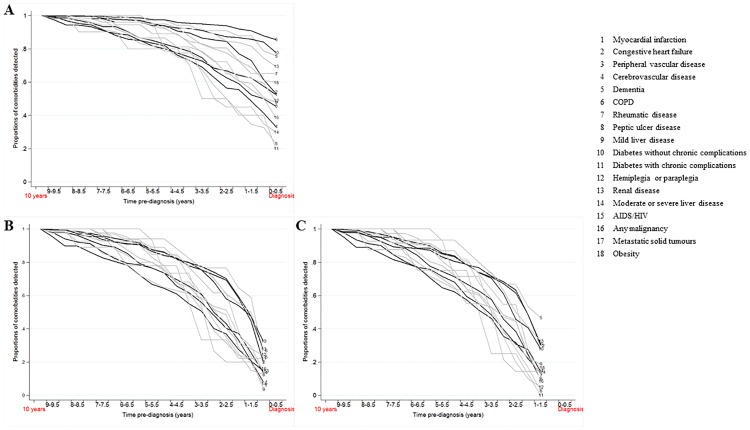
Proportions of individual comorbidities recorded for laryngeal cancer patients diagnosed in 2013 for increasing time periods pre-diagnosis, N = 1,789. 1: Myocardial infarction; 2: Congestive heart failure; 3: Peripheral vascular disease; 4: Cerebrovascular disease; 5: Dementia; 6: COPD; 7: Rheumatic disease; 8: Peptic ulcer disease; 9: Mild liver disease; 10: Diabetes without chronic complications; 11: Diabetes with chronic complications; 12: Hemiplegia or paraplegia; 13: Renal disease; 14: Moderate or severe liver disease; 15: AIDS/HIV; 16: Any malignancy; 17: Metastatic solid tumours; 18: Obesity.

Fig 2 depicts time-varying proportions of comorbidity according to backward availability of HES data. The x-axis represents time from 10 years before the diagnosis of laryngeal cancer (2003) to the diagnosis time in 2013. We considered that, at 10 years of retrospective follow-up, we have reached the maximum number of comorbidities identifiable from HES, and prevalent in the patient population. From the time at diagnosis, the cumulative proportions of all possible comorbidities converged to 100%. Each comorbidity that composes the CCI is represented by a curve of the time-varying cumulative proportion. No restriction window was included in Fig 2A while Fig 2B and 2C illustrate the impact of a six-month and 12-month restriction window, respectively. The cumulative proportions of any comorbidities reach 80% and above soon after six years before the diagnosis: six years with no restriction window, six and a half years with a six-month restriction and seven years with a 12-month restriction: an overall window of six years already captures the vast majority of comorbidities there are to report in the ten years preceding the diagnosis.

The proportion of patients with COPD (Chronic Obstructive Pulmonary Disease) is high immediately before diagnosis and does not increase greatly with additional years, approximately 15%, reflecting that more than 85% of COPD is identified in the six months before the diagnosis. In Fig 2B and 2C, that proportion is restricted to around 30%. Furthermore, using a restriction window make all cumulative incidence curves follow the same pattern: between 0 and 40% of the final proportions of comorbidities are detected 6 or 12 months before diagnosis. The cumulative proportions of comorbidities increase at an approximate similar rate reaching 100% at ten years.

We provide absolute and relative measures of the impact of applying restriction windows on the assessment of comorbidities. Among the 1,789 laryngeal cancer patients diagnosed in 2013, 51% present with at least one comorbid condition. That proportion drops to 34% and 32% if a six- or 12-month restriction window is applied. It highlights that 17% of these comorbidities are first reported in the six months preceding the diagnosis (Table 2).

**Table 2:** Numbers and proportions of patients with comorbidities detected at selected time points through the ten years preceding laryngeal cancer diagnosis made in 2013, given three restriction windows, N = 1,789.

	No restriction window		6-month restriction window		12-month restriction window	
Time (years)	N.	prop.	N.	prop.	N.	prop.
1	755	0.42	145	0.08		
2	807	0.45	321	0.18	234	0.13
3	839	0.47	415	0.23	345	0.19
4	860	0.48	468	0.26	405	0.23
5	878	0.49	524	0.29	474	0.26
6	892	0.50	541	0.30	493	0.28
7	905	0.51	569	0.32	522	0.29
8	912	0.51	591	0.33	546	0.31
9	918	0.51	609	0.34	566	0.32
10*	918	0.51	614	0.34	572	0.32

*10 years is the maximum number of years we could screen for comorbidities

Overall, the reliability of the recording of comorbidities in HES is moderate for 7 comorbidities with an ICC ranging between 0.3 for rheumatic disease and 0.62 for dementia. All other comorbidities showed ICCs less than 0.3 for the 2013 laryngeal cancer cohort indicating weak internal reliability (Table 3). The ICC for the 2013 laryngeal cancer cohort and all the available data (cohorts from 2005 to 2013, N = 16,112) were similar, which indicated the absence of secular trends. Dementia, COPD, diabetes without chronic complication and renal disease showed a moderate ICC (≥0.5) while peptic ulcer and myocardial infarction consistently showed a lower ICC. In evaluating external reliability, we found that the proportion of agreement between ONS and HES was notably high (86.3%).

**Table 3:** Reliability of HES data; Internal reliability: Intra-class correlation coefficient for each comorbidity; external reliability: Proportion of agreement for the diagnosis of the index cancer between the diagnostic fields of HES and the cancer registration data, by cancer and year of diagnosis.

	Larynx (2013)*			Larynx**		
		95% CI			95% CI	
Internal reliability	ICC	low	high	ICC	low	high
Myocardial infarction	0.27	0.17	0.37	0.27	0.24	0.30
Congestive heart failure	0.33	0.21	0.44	0.39	0.35	0.44
Peripheral vascular disease	0.26	0.16	0.35	0.34	0.31	0.38
Cerebrovascular disease	0.29	0.20	0.38	0.33	0.30	0.36
Dementia	0.56	0.32	0.80	0.62	0.53	0.72
Chronic pulmonary disease	0.38	0.31	0.45	0.54	0.52	0.56
Rheumatic disease	0.30	0.11	0.50	0.50	0.42	0.57
Peptic ulcer disease	0.09	0.01	0.18	0.19	0.15	0.22
Mild liver disease	0.31	0.20	0.43	0.39	0.34	0.44
Diabetes without chronic complication	0.38	0.28	0.48	0.52	0.48	0.56
Diabetes with chronic complication	0.18	-0.06	0.41	0.42	0.31	0.53
Hemiplegia or paraplegia	0.29	0.12	0.46	0.34	0.27	0.42
Renal disease	0.19	0.07	0.31	0.49	0.44	0.54
Any malignancy, including lymphoma and leukemia, except malignant neoplasm of skin	0.29	0.01	0.58	0.35	0.27	0.43
Moderate or severe liver disease	0.31	0.10	0.53	0.28	0.20	0.36
Metastatic solid tumor	0.00	0.00	0.00	0.00	0.00	0.00
AIDS/HIV	0.25	-0.88	1.39	0.43	-0.10	0.96
Morbid obesity	0.18	0.06	0.31	0.30	0.24	0.36
**External reliability**						
Proportion agreement HES/ONS	86.31	84.62	87.87	86.26	85.72	86.79

*Larynx 2013-cohort, N = 1,789

** All cohorts, N = 16,112

Table 4 shows the effect of cancer-related and non-cancer-related COPD on cancer mortality. COPD was defined as related to the cancer if first diagnosed within either 6, 12 or 24 months before the cancer diagnosis. For all three intervals, age-adjusted non-cancer related comorbidities were associated with higher odds of cancer mortality: hazard ratios were 1.26 (CI: 0.98–1.63), 1.26 (CI: 0.97–1.63) and 1.16 (CI: 0.86–1.56) for comorbidities assessed 6, 12 or 24 months away from the cancer diagnosis, respectively. Likewise, cancer-related comorbidities were consistently associated with a higher cancer mortality risk with all hazard ratios over 1.5. However, in multivariate adjusted Cox models where we included age, cancer- and non-cancer related comorbidities as independent predictors, the point estimate for the effect of non-cancer related comorbidities was reversed (HR: 0.98, CI: 0.72–1.32; 0.95, CI: 0.70–1.28; 0.86, CI: 0.62–1.19 for all three intervals assessed). Despite the lack of statistical significance the change in the point estimates indicate a possible bias.. S1 Fig illustrates the collider effect that cancer-related comorbidities (W2) might have on non-cancer related comorbidities (W1). Cancer related comorbidities (W2) acts as a collider that opens the backdoor path between unmeasured confounders associated with both non-cancer related comorbidities and the outcome (Y).

**Table 4:** Hazard Ratios (HR) for the effects of cancer-related* and non-cancer related* comorbidities on the overall hazard of death, laryngeal cancer patients diagnosed in England in 2013, N = 1,789.

	COPD					
	Cancer-related			Non-cancer related		
*Restriction window*	HR	95% CI		HR	95% CI	
***6 months***	0–6 months			>6 months		
M0				1.26	0.98	1.63
M1	1.50	1.21	1.86			
Full model	1.51	1.18	1.95	0.98	0.72	1.32
***12 months***	0–12 months			>12 months		
M0				1.26	0.97	1.63
M1	1.55	1.25	1.92			
Full model	1.59	1.24	2.03	0.95	0.70	1.28
***24 months***	0–24 months			>24 months		
M0				1.16	0.86	1.56
M1	1.56	1.26	1.92			
Full model	1.64	1.30	2.07	0.86	0.62	1.19

M0: model including non-cancer related comorbidities, adjusted for age. M1: model including cancer-related comorbidities, adjusted for age. Full model: model including both variables for comorbidities, adjusted for age.

* Cancer related comorbidities are those first recorded in the restrictions windows of 6, 12 or 24 months immediately preceding the cancer diagnosis; non-cancer related comorbidities are those recorded in the ten years preceding the cancer diagnosis, excluding the restriction window.

## Discussion

This study highlights the importance of explicitly stating and empirically proving the assumptions made in the assessment of cancer comorbidities using administrative health data. We recommend considering time as an important confounder in the assessment of comorbidities by defining an optimal window and a restriction window. Furthermore, consistency of the optimal window ensures there is no selection bias associated with time, as all patients included in the incident cancer data have the same follow-up period to be assessed for comorbidities. We demonstrate that a 6-year window is an optimal period for identifying comorbidities in our setting. The purpose of the restriction window is to prevent paradoxical effects when assessing the impact of comorbidities on cancer survival. With empirical evidence we highlight the need for a restriction window of at least six months prior to laryngeal cancer diagnosis, when we consider the effect of COPD. Such exercise would need to be repeated for different combinations of cancer sites and comorbidities. Additionally, the code for the computing algorithm is available as proof of reproducible research (S1 Code).

The number of studies in cancer epidemiology using derived information of comorbidities from administrative or clinical data has grown in the last five years.[10, 22–26] There is a wealth of literature on comorbidity scores [23, 27, 28] and on adapting them to different data sets [29–31], varying numbers of comorbidities included for consideration, and varying subsets of the population [32–34] or diseases of interest.[35, 36] The literature mostly focusses on how administrative data compares to medical records [22] in terms of identifying relevant comorbidities, and if a particular score or modified score is a good predictor of mortality. [37, 38] However, there is no clear consensus on how to assess and estimate comorbidities using administrative or other type of health data. Furthermore, the majority of recent research analysing comorbidity data do not state major assumptions made to derive information on comorbidity; criteria such as validity and reliability are not routinely assessed.[20, 22–34] In epidemiological studies any assumption made during the data generation process and analysis must be stated. [39] Therefore, the first step to develop a uniform approach to assess and use comorbidities in future studies is to state the assumptions made to generate the data. We explicitly document and empirically prove the set of assumptions needed to derive comorbidity information from secondary care health administrative data.

Time is one of the most important confounders in epidemiology. Given patients with larger follow-up period might show higher probability of identifying comorbidities, we set an optimal window so that the assessment of comorbidities is independent of time (i.e., securing the same follow-up time for comorbidities for all cancer patients). Furthermore, the optimal window helps to maximise the equal number of years that all cancer patients included in the analysis were followed up.[40] Following the weak ICC for hospital administrative data presented here for many of the comorbidities assessed, there is a rational for using the longest possible assessment window in order to maximise the detection of existing comorbidities. An audit of HES codes showed 90.5% accuracy for identifying 8 major comorbidities, indicating that HES diagnostic fields can confidently predict the actual presence of the comorbidities. Improving the protocol for documenting comorbidities with clinicians and providing further training to administrative clerks could enhance the assessment of comorbidities using HES.[41]

We also compared the prevalence of comorbid conditions in our laryngeal cancer population with that of the general population: comorbidities sharing the same risk factors as laryngeal cancer were much more prevalent in our data, while all other comorbidities were comparable to published prevalence for the general population (data not shown). These results are in agreement with the HES clinical audit.[41]

Some studies have used a restriction window to assess the effect of comorbidities on cancer survival, although the assumptions made to set this window have not been explicitly justified or documented.[40, 42] To our knowledge, we are the first to empirically show the impact of neglecting this principle. A paradoxical association between cancer-related comorbidities and mortality, such as obtaining a protective effect from a risk factor (COPD) known to predict the outcome (mortality due to laryngeal cancer), occurs if a restriction window is not set. This paradoxical association occurs when the probability of the exposure is associated with the outcome being studied.[15] Likewise, a collider stratification bias may occur when we condition on a common effect of exposure and outcome, i.e. non-cancer related comorbidities conditioned on cancer-related comorbidities and cancer.[43] Therefore, when the interest of researchers is to explain the effect of comorbidities on cancer survival, we advise epidemiologists to think carefully about the particular effect of individual comorbidities on specific cancer sites to avoid reporting spurious or paradoxical protective effects of comorbidities.[44–46]

We show that there are fewer differences between a 6- and 12-month restriction window than between no window and a 6-month window, mostly related to cancer-related comorbidities recorded for the first time in the 6 or 12 months before the cancer diagnosis. This finding highlights the potential for earlier cancer diagnosis. In particular, the high proportion of COPD could reflect a mis-diagnosis of laryngeal cancer.

Despite documented differences between administrative data and medical records[47], both types of data produce comorbidity scores that have similar predictive power.[48, 49] We found over 86% agreement between the HES data and Cancer Registrations for the registration of laryngeal cancer. Other limitations include the necessary computing resources for handling big data, the availability of data for the assessment of comorbidities (2003 to 2015), the relatively small number of comorbidities we focused on, and the external validity of our findings limited to hospital records in England. However, our approach is general, and it could be valid in other settings. We recognise we are missing some lifestyle risk factors such as tobacco smoking, alcoholism, drug abuse and other conditions such as asthma, eating disorders and epilepsy, which would undoubtedly impact outcomes.

We encourage researchers to consider our recommendations for the assessment and use of comorbidities. We have clarified the set of assumptions used to identify cancer patients’ comorbidities using hospital data. Moreover, we have demonstrated our assumptions through empirical analyses based on current epidemiologic knowledge. Our algorithm for the assessment of comorbidities could be considered as a state-of-the-art method for the evaluation of comorbidities using administrative health data in population-based cancer research epidemiology. Furthermore, we have shown that administrative hospital data is a valuable and consistent source of information allowing population-based cancer researchers to update comorbidities information for patients.

## Supporting information

S1 Code STATA code. (DOCX)

S1 Fig The direct acyclic graph for the collider stratification bias from non-cancer related comorbidities. W1: Non-cancer related comorbidities; W2: Cancer-related comorbidities; W3: Age; Y: Death.
(XLSX)

S1 Table Comorbidities and their ICD-10 codes, as defined for the Charlson comorbidity index, and obesity. (XLSX)
