# Supplementary material for: Reproducibility, reliability and validity of population-based administrative health data for the assessment of cancer non-related comorbidities
Source: PLoS One. 2017 Mar 6;12(3):e0172814. doi: 10.1371/journal.pone.0172814 (PMC5338773; doi:10.1371/journal.pone.0172814)
Supplement: S1 Code — (DOCX) [file pone.0172814.s001.docx]

**S1 Code.**

1. **STATA code for creating comorbidity-specific binary variables**

The programme provided below produces a matrix of 18 column vectors consisting of binary indicators of the presence of the 17 Charlson index comorbid conditions and morbid obesity. The code is adapted to extract ICD-10 codes from the 14 column vectors containing the diagnostic information for individual hospital episodes. The code should be saved as a Stata **.do** file**,** named **“BinaryComorbidities.do”** to be used later**.**

The code is based on the seminal article published by Quan H. et al. in 2005. (Quan H. et al. Coding Algorithms for defining comorbidity in ICD-9-CM and ICD-10 Administrative Data. Medical Care. Volume 43, Number 11, November 2005).

**Note**: Stata code is highlighted in red.

**Commented code:**

**** 1. create binary variables that will contain information on comorbidities**

forval i in 1/18{

gen cm`i’= 0

}

**** 2. assess for the presence of each comorbidity (1-18) in the 14 diagnostic fields (diag_1 – diag_14)**

forval i = 1(1)14 {

replace cm1 = 1 if cm1 == 0 ///

& (substr(diag_`i',1,3)=="I21" | substr(diag_`i',1,3)=="I22" | diag_`i'=="I252")

replace cm2 = 1 if cm2 == 0 ///

& (substr(diag_`i',1,3)=="I43" | substr(diag_`i',1,3)=="I50" | /// diag_`i'=="I099" | diag_`i'=="I110" | ///

diag_`i'=="I130" | diag_`i'=="I132" | diag_`i'=="I255" | /// diag_`i'=="I420" | diag_`i'=="I425" | diag_`i'=="I426" | ///

diag_`i'=="I427" | diag_`i'=="I428" | diag_`i'=="I429" | /// diag_`i'=="P290")

replace cm3 = 1 if cm3 == 0 ///

& (substr(diag_`i',1,3)=="I70" | substr(diag_`i',1,3)=="I71" | /// diag_`i'=="I731" | diag_`i'=="I738" | ///

diag_`i'=="I739" | diag_`i'=="I771" | diag_`i'=="I790" | /// diag_`i'=="I792" | diag_`i'=="K551" | diag_`i'=="K558" | ///

diag_`i'=="K559" | diag_`i'=="Z958" | diag_`i'=="Z959")

replace cm4 = 1 if cm4 == 0 ///

& (substr(diag_`i',1,3)=="G45" | substr(diag_`i',1,3)=="G46" | /// diag_`i'=="H340" | substr(diag_`i',1,3)=="I60" | ///

substr(diag_`i',1,3)=="I61" | substr(diag_`i',1,3)=="I62" | /// substr(diag_`i',1,3)=="I63" | substr(diag_`i',1,3)=="I64" | ///

substr(diag_`i',1,3)=="I65" | substr(diag_`i',1,3)=="I66" | /// substr(diag_`i',1,3)=="I67" | substr(diag_`i',1,3)=="I68" | ///

substr(diag_`i',1,3)=="I69")

replace cm5 = 1 if cm5 == 0 ///

& (substr(diag_`i',1,3)=="F00" | substr(diag_`i',1,3)=="F01" | /// substr(diag_`i',1,3)=="F02" | substr(diag_`i',1,3)=="F03" | ///

diag_`i'=="F051" | diag_`i'=="G311" | substr(diag_`i',1,3)=="G30")

replace cm6 = 1 if cm6 == 0 ///

& (diag_`i'=="I278" | diag_`i'=="I279" | substr(diag_`i',1,3)=="J40" | substr(diag_`i',1,3)=="J41" | diag_`i'=="J42" | ///

substr(diag_`i',1,3)=="J43" | substr(diag_`i',1,3)=="J44" | /// substr(diag_`i',1,3)=="J45" | substr(diag_`i',1,3)=="J46" | ///

substr(diag_`i',1,3)=="J47" | substr(diag_`i',1,3)=="J60" | /// substr(diag_`i',1,3)=="J61" | substr(diag_`i',1,3)=="J62" | ///

substr(diag_`i',1,3)=="J63" | substr(diag_`i',1,3)=="J64" | /// substr(diag_`i',1,3)=="J65" | substr(diag_`i',1,3)=="J66" | ///

substr(diag_`i',1,3)=="J67" | diag_`i'=="J684" | diag_`i'=="J701" | diag_`i'=="J703")

replace cm7 = 1 if cm7 == 0 ///

& (substr(diag_`i',1,3)=="M05" | substr(diag_`i',1,3)=="M06" | diag_`i'=="M315" | substr(diag_`i',1,3)=="M32" | ///

substr(diag_`i',1,3)=="M33" | substr(diag_`i',1,3)=="M34" | diag_`i'=="M351" | diag_`i'=="M353" | diag_`i'=="M360")

replace cm8 = 1 if cm8 == 0 ///

& (substr(diag_`i',1,3)=="K25" | substr(diag_`i',1,3)=="K26" | substr(diag_`i',1,3)=="K27" | substr(diag_`i',1,3)=="K28")

replace cm9 = 1 if cm9 == 0 ///

& (substr(diag_`i',1,3)=="B18" | diag_`i'=="K700" | diag_`i'=="K701" | diag_`i'=="K702" | diag_`i'=="K703" | ///

diag_`i'=="K709" | diag_`i'=="K713" | diag_`i'=="K714" | /// diag_`i'=="K715" | diag_`i'=="K717" | substr(diag_`i',1,3)=="K73"///

| substr(diag_`i',1,3)=="K74" | diag_`i'=="K760" ///

| diag_`i'=="K762" | diag_`i'=="K763" | diag_`i'=="K764" | ///

diag_`i'=="K768" | diag_`i'=="K769" | diag_`i'=="Z944")

replace cm10 = 1 if cm10 == 0 ///

& (diag_`i'=="E100" | diag_`i'=="E101" | diag_`i'=="E106" | /// diag_`i'=="E108" | diag_`i'=="E109" | ///

diag_`i'=="E110" | diag_`i'=="E111" | diag_`i'=="E116" | /// diag_`i'=="E118" | diag_`i'=="E119" | ///

diag_`i'=="E120" | diag_`i'=="E121" | diag_`i'=="E126" | /// diag_`i'=="E128" | diag_`i'=="E129" | ///

diag_`i'=="E130" | diag_`i'=="E131" | diag_`i'=="E136" | /// diag_`i'=="E138" | diag_`i'=="E139" | diag_`i'=="E140" | ///

diag_`i'=="E141" | diag_`i'=="E146" | diag_`i'=="E148" | /// diag_`i'=="E149")

replace cm11 = 1 if cm11 == 0 ///

& (diag_`i'=="E102" | diag_`i'=="E103" | diag_`i'=="E104" | /// diag_`i'=="E105" | diag_`i'=="E107" | ///

diag_`i'=="E112" | diag_`i'=="E113" | diag_`i'=="E114" | /// diag_`i'=="E115" | diag_`i'=="E117" | ///

diag_`i'=="E122" | diag_`i'=="E123" | diag_`i'=="E124" | /// diag_`i'=="E125" | diag_`i'=="E127" | ///

diag_`i'=="E132" | diag_`i'=="E133" | diag_`i'=="E134" | /// diag_`i'=="E135" | diag_`i'=="E137" | diag_`i'=="E142" | ///

diag_`i'=="E143" | diag_`i'=="E144" | diag_`i'=="E145" | /// diag_`i'=="E147")

replace cm12 = 1 if cm12 == 0 ///

& (diag_`i'=="G041" | diag_`i'=="G114" | diag_`i'=="G801" | /// diag_`i'=="G802" | ///

substr(diag_`i',1,3)=="G81" | substr(diag_`i',1,3)=="G82" | /// diag_`i'=="G830" | diag_`i'=="G831" | ///

diag_`i'=="G832" | diag_`i'=="G833" | diag_`i'=="G834" | /// diag_`i'=="G839")

replace cm13 = 1 if cm13 == 0 ///

& (diag_`i'=="I120" | diag_`i'=="I131" | diag_`i'=="N032" | /// diag_`i'=="N033" | ///

diag_`i'=="N034" | diag_`i'=="N035" | diag_`i'=="N036" | /// diag_`i'=="N037" | diag_`i'=="N052" | ///

diag_`i'=="N053" | diag_`i'=="N054" | diag_`i'=="N055" | /// diag_`i'=="N056" | diag_`i'=="N057" | ///

substr(diag_`i',1,3)=="N18" | substr(diag_`i',1,3)=="N19"| /// diag_`i'=="N250" | diag_`i'=="Z490" | ///

diag_`i'=="Z491" | diag_`i'=="Z492" | diag_`i'=="Z940" | /// diag_`i'=="Z992")

replace cm14 = 1 if cm14 == 0 ///

& (diag_`i'=="I850" | diag_`i'=="I859" | diag_`i'=="I864" | /// diag_`i'=="I982" | ///

diag_`i'=="K704" | diag_`i'=="K711" | diag_`i'=="K721" | /// diag_`i'=="K729" | diag_`i'=="K765" | diag_`i'=="K766" | ///

diag_`i'=="K767")

replace cm15 = 1 if cm15 == 0 ///

& (substr(diag_`i',1,3)=="B20" | substr(diag_`i',1,3)=="B21" | /// substr(diag_`i',1,3)=="B22" | substr(diag_`i',1,3)=="B24")

****** 3. Cancer-related comorbidities, from HES information only. It can be removed/completed by cancer registration data**

gen d = substr(diag_`i',2,3)

destring d, replace force

replace cm16 = 1 if cm16 == 0 ///

& ((substr(diag_`i',1,1)=="C" & d <=26 & d != .) | (substr(diag_`i',1,1)=="C" & d >=30 & d<=34 & d != . ) | ///

(substr(diag_`i',1,1)=="C" & d >=37 & d<=41 & d != . ) | /// substr(diag_`i',1,3)=="C43" | ///

(substr(diag_`i',1,1)=="C" & d >=45 & d<=58 & d != . ) | /// (substr(diag_`i',1,1)=="C" & d >=60 & d<=76 & d != . ) | ///

(substr(diag_`i',1,1)=="C" & d >=81 & d<=85 & d != . ) | /// substr(diag_`i',1,3)=="C88" | (substr(diag_`i',1,1)=="C" & ///

d >=90 & d<=97 & d != . ))

replace cm17 = 1 if cm17 == 0 ///

& (substr(diag_`i',1,1)=="C" & d >=77 & d<=80 & d != . )

drop d

****** 4. Obesity**

replace cm18 = 1 if cm18 == 0 & substr(diag_`i',1,3) == "E66"

}

1. **Algorithm used to develop the code for the assessment of comorbidities in 6-monthly intervals**

**Note**: Stata code is highlighted in red.

**1. Calculate time spanned from cancer diagnosis to the start of the hospital episode**

- gen length time between diagnosis date and episode start date

**** Formatting dates: make sure all dates are month, day, year (**mdy**) format**

- - **Date of diagnosis**

gen datdiag=date(subinstr(diagmdy,"-","",.),"YMD")

format datdiag %td

- **Date of start of episode**

* start of episode date (in-patient format)

* arrival date (out-patient and accident and emergency format)

replace datepistart = arrivaldatemdy if dataset_flag == 3

replace datepistart = apptdatemdy if dataset_flag == 2

**** Calculate length of time between each episode and date of diagnosis**

gen length = datepistart - datdiag

Ignore episodes that were recorded prior to 1997:

- reason 1: ICD-9 and earlier versions used
- reason 2: may be wrong dates as HES only started in 1997

replace length = . if year(datepistart)<1997

- for each patient, sort by decreasing length: episodes closest to diagnosis first down to episodes furthest to diagnosis

gsort id -length

by id: gen n=_n

**** Save dataset as a temporary file so we get the information back for those who do not have any comorbidities recorded**

tempfile origData

save "`origData'", replace

**** Drop episodes after diagnosis date**

drop if length>0

**2.** **Create dummy binary variables for each comorbidity in each episode independently using the do file from part A**

do "BinaryComorbidities.do"

You will obtain for each comorbidity 18 new variables, namely:

**cm`i'** where `i’ refers to the number assigned to each comorbidity from 1 to 18: a binary indicator of whether a comorbid condition is present before the cancer diagnosis for each patient

**3. Earliest date at which a comorbidity is recorded:**

- Loop on each comorbidity (1 to 18): i = 1-18
- For each patient, get the minimum length (i.e. earliest time, since length has only negative values) that a comorbidity is recorded in hospital data

gsort id -length n

by id: egen minL`i' = min(length) if cm`i' == 1

by id: gen CM`i' = 1 if minL`i' != .

by id: gen CM_date_`i' = datepistart if CM`i' == 1 & length == minL`i'

- repeat the information just created on all rows for a given patient

bysort id (CM`i'): replace CM`i' = CM`i'[1]

bysort id (CM_date_`i'): replace CM_date_`i' = CM_date_`i'[1]

format CM_date_`i' %td

bysort id (minL`i'): replace minL`i' = minL`i'[1]

- keep a unique record on patient and first dates in a temporary dataset:

save "`dates'", replace

**4. Assessing comorbidities in 6-monthly time periods**

- Split time into 6-monthly intervals in the ten years prior to diag, from 1 to 21
- Loop on each interval (1 to 21): inter = 1-21

**preserve**

* we keep episodes in each six-month interval prior to diagnosis

drop if Inter6m != `inter'

gsort id -length n

* Loop on each comorbidity (1 to 18): i = 1-18

* we check whether comorbidity `i' is recorded in that interval:

by id: gen sum`inter'_cm`i' = sum(cm`i')

by id: gen CM_`inter'_`i' = cond(sum`inter'_cm`i'>0,1,0) if _n == _N

label var CM_`inter'_`i' "CM`i' in `inter' interval before diag"

keep id CM_`inter'_*

* keep only the last row, where the useful info (CM_`inter'_*) is kept, all other rows have missing CM_`inter'_*

drop if CM_`inter'_1 == .

sort id

* we save a temporary file ``inter'' for each interval that contains the useful information on all comorbidities

save "``inter''", replace

**restore**

* back to the original dataset

use "`origData'", clear

**5. Loop on each interval (1 to 21): inter = 1-21**

sort id

- merge each interval-specific dataset "``inter''" on to the original dataset and keep merged and original records
- merge dataset "`dates'" containing information on earliest dates for each comorbidity on to the newly created dataset and keep merged and original records.

1. **Program to group information from six-monthly intervals into bigger intervals**

**Note**: Stata code is highlighted in red.

1. **Program**

cap program drop CMbyTime

program define CMbyTime

while "`1'" != "" {

* Start and end are the input of the user. They represent the time period in which the user wants to look at comorbidities

local start = substr("`1'",1,strpos("`1'","_")-1)

local end = substr("`1'",strpos("`1'","_")+1,length("`1'"))

local start_nb = (`start'/6)+1

local end_nb = `end'/6

* For each comorbidity, we combine the information from the six-month intervals that compose the overall time-period: cm = 1(1)18

gen CM_HES_`1'_cm`cm' = CM_HES_`start_nb'_`cm'

local j = `start_nb'+1

* While we are still within the overall time-period we add information on individual comorbidities:

`j' <= `end_nb'

replace CM_HES_`1'_cm`cm' = CM_HES_`1'_cm`cm'+CM_HES_`j'_`cm'

local j = `j'+1

* The final variable is a binary variable

replace CM_HES_`1'_cm`cm' = cond(CM_HES_`1'_cm`cm'>0,1,0)

* Charlson comorbidity index for that overall time-period

gen CCI_HES_`1' = 1*(CM_HES_`1'_cm1+CM_HES_`1'_cm2+CM_HES_`1'_cm3+CM_HES_`1'_cm4+CM_HES_`1'_cm5+CM_HES_`1'_cm6+CM_HES_`1'_cm7+CM_HES_`1'_cm8+CM_HES_`1'_cm9) ///

+ 2*(CM_HES_`1'_cm10+CM_HES_`1'_cm11+CM_HES_`1'_cm12+CM_HES_`1'_cm13+CM_HES_`1'_cm16) ///

+ 3*(CM_HES_`1'_cm14) ///

+ 6*(CM_HES_`1'_cm15+CM_HES_`1'_cm17)

mac shift

}

end

1. **Call program**

* use your data, in which you have the 18 comorbidity fields for each six-month intervals

* Select the cohorts of patients so that they can all be followed up for the overall period you have in mind (here as an illustration we look at 12 months to 6 years prior to cancer diagnosis)

* Call the program with

CMbyTime "12_72"

This is it!

Congratulations!
